# Glomerular diseases after immune checkpoint inhibitors use: What do We know so far?

**DOI:** 10.1080/0886022X.2022.2147439

**Published:** 2022-11-24

**Authors:** Xue He, Fei Liu, Yanyan Jin, Haidong Fu, Jianhua Mao

**Affiliations:** Department of Nephrology, The Children's Hospital, Zhejiang University School of Medicine, National Clinical Research Center for Child Health, National Children's Regional Medical Center, Hangzhou, Zhejiang, China

**Keywords:** Immune checkpoint inhibitors, glomerular disease, glomerulonephritis, renal vasculitis, minimal change disease

## Abstract

The research and application of immune checkpoint inhibitors (ICIs) have enormously promoted the progression of tumor treatment. Gradual implementation of ICIs in clinical practice is largely limited as they exert uncontrolled collateral effects on the immune system, such as immune-related adverse events (irAEs); this includes rarely reported glomerular diseases. This study aimed to describe the clinical and pathological manifestation of ICIs-induced glomerular diseases and focused on the mechanism and therapeutic strategy for glomerular diseases associated with ICIs. The data of 53 patients with glomerular diseases related to ICIs were retrieved from the PubMed database. The most frequently reported ICIs-related glomerular diseases were pauci-immune glomerulonephritis (28.3%), podocytopathies (26.4%), and immune-complex glomerulonephritis (18.9%). Moreover, anti-PD1 antibodies were the most commonly used ICIs (71.4%). Most patients receiving ICIs discontinued the treatment (89.4%) and were initiated with steroids (87.2%). Rituximab was also useful in the treatment, especially for renal vasculitis. Rechallenging ICIs could be considered for cancer progression or as salvage therapy, where rechallenging ICI therapy with steroids may be beneficial. We believe the treatment should be personalized based on the degree of renal pathology, serum creatinine (Scr), and tumor progression to obtain a good prognosis.

## 1. Introduction

In the past decade, the exploration and application of ICIs have greatly promoted tumor treatment progression. ICIs are monoclonal antibodies targeting inhibitory receptors that block the co-inhibitory molecules expressed on T cells and tumor cells for improving anti-tumor immunity [1]. These inhibitory receptors include the cytotoxic T lymphocyte-associated antigen 4 (CTLA-4), the programmed death 1 protein (PD-1), and the programmed death-ligand 1 (PD-L1). ICIs can inhibit CTLA-4 and PD-1/PD-L1 to enhance tumor-directed immune responses and have been used extensively as a novel therapeutic agent to treat solid and hematologic malignancies, especially for advanced melanoma. About 50% of patients with advanced melanoma have shown tumor regression, thereby suggesting that cancer can be controlled durably [2–4]. ICIs have now been approved for several diseases such as melanoma, Hodgkin’s lymphoma, small cell and non-small cell lung cancer, renal cell carcinoma, and urothelial carcinoma [5]. At present, the approved ICIs mainly contain ipilimumab (anti-CTLA-4 antibodies), nivolumab, pembrolizumab (anti-PD-1 antibodies), durvalumab, and atezolizumab (anti-PD-L1 antibodies) [1].

The side effects of ICIs have been demonstrated with the increasing use of ICIs [6]. The incidence of irAEs ranges from 60% to 85%, generally with mild to moderate grades. For irAEs, nonspecific immune activation against self-antigens may help mediate their pathophysiology, thereby impacting common organs such as the skin, gastrointestinal tract, and liver [7,8]. The incidence of renal irAEs is lower compared to the involvement of other organs, with a range of 1.4%–4.9% [9]. Although tubulointerstitial nephritis (TIN) is a common renal lesion resulting from ICIs, reports of various glomerular diseases have increased in recent years, involving renal vasculitis, minimal change disease (MCD), membranous nephropathy (MN), and IgA nephropathy (IgAN) [10–12]. Kidney injury might result in various sequelae and possibly restrict the further treatment options of oncology. Hence, timely follow-up and treatment are necessary. Our study primarily focuses on glomerular diseases subsequent to the use of ICIs, which are rarer and more severe. Considering that the first case of ICI-related glomerular diseases was reported in 2009, a search of the PubMed database was performed for various literature from January 2009 to December 2021, with the results enriched by manual searches and citation mining. Notably, we conducted a review of reported cases regarding glomerular diseases related to ICI therapy to characterize the clinical features and described the probable mechanism, treatment, and management strategies.

## 2. Clinical features and pathology

A total of 33 articles were retrieved, including individual case reports and serial reports. These articles consisted of data from 53 patients with glomerular disease related to ICIs therapy (CTLA-4, PD-1, and PD-L1 pathway agents) [10,11,13–43]. This study excluded articles that did not report new and specific cases regarding glomerular diseases. Information collected include age, gender, cancer diagnosis, ICI name and class, Scr, routine urine test, serologic findings, kidney biopsy findings, treatment therapy, and outcomes.

Classification of glomerular diseases and glomerulonephritis (GN) was carried out according to the KDIGO 2021 Guideline for the Management of Glomerular Diseases [44] and the Mayo Clinic/Renal Pathology Society Consensus Report [45].

The malignancies involved consisted of melanoma (32.6%), non-small cell lung cancer (NSCLC, 26.5%), renal cell carcinoma (RCC, 12.2%), gastrointestinal cancer (6.1%), lymphoma (6.1%), and malignant pleural mesothelioma. The mean age of the patients was 63.78 ± 1.61. The majority of patients were male (76.1%), owing to the high prevalence rate of skin cancer or lung cancer in males. Widely used ICIs were anti-PD1 antibodies (71.4%). Glomerular diseases associated with the use of ICIs therapy included 15 cases of renal pauci-immune GN (28.3%), 14 cases of podocytopathies (26.4%), 10 cases of immune-complex GN (18.9%), 4 cases of MN (7.5%), 4 cases of AA amyloidosis (7.5%), 4 cases of C3 glomerulopathy (7.5%), and 2 cases of anti-glomerular basement membrane GN (3.8%) (Table 1). Most of them were accompanied by renal functional lesions, with clinical manifestations such as proteinuria, hematuria, pyuria, oliguria, edema, and hypertension.

**Table 1.** Characteristics of reported patients with glomerular disease associated with ICIs.

Characteristics	N (%)
Total number	53
Sex	
Male	35 (76.1%)
Femal	11 (23.9%)
Not available	7
Tumor types	
Melanoma	16 (32.6%)
Non-small cell lung cancer	13 (26.5%)
Renal cell carcinoma	6 (12.2%)
Gastrointestinal cacer	3 (6.1%)
Lymphoma	3 (6.1%)
Others	8 (16.3%)
Not available	4
ICIs therapy	
Anti- PD1 antibodies	35 (71.4%)
*Nivolumab*	18 (36.7%)
*Pembrolizumab*	15 (30.6%)
*Tislelizumab*	1 (2.0%)
*SHR-1210*	1 (2.0%)
Anti-PDL1 antibodies	2 (4.1%)
*Durvalumab*	2 (4.1%)
Anti-CTLA4 antibodies	4 (8.1%)
*Ipilimumab*	3 (6.1%)
*Tremelimumab*	1 (2.0%)
Combination treatments	8 (16.3%)
Not available	4
Renal pathology	
Pauci-immune GN	15 (28.3%)
*ANCA positive*	4 (7.5%)
*ANCA negative or undetected*	11 (20.8%)
Podocytopathies	14 (26.4%)
*MCD*	12 (22.6%)
*FSGS*	2 (3.8%)
Immune-complex GN	10 (18.9%)
*IgA nephropathy*	6 (11.3%)
*Others*	4 (7.5%)
AA amyloidosis,	4 (7.5%)
Membranous nephropathy	4 (7.5%)
C3 glomerulopathy	4 (7.5%)
Anti-glomerular basement membrane GN	2 (3.8%)
Treatment	
ICIs discontinued	42 (89.4%)
Steroids	41 (87.2)%
High dose methylprednisolone	11 (23.4%)
Cyclophosphamide	2 (4.3%)
Rituximab	7 (14.9%)
Mycophenolate	3 (6.4%)
Infliximab	2 (4.3%)
Tocilizumab	1 (2.1%)
TNFα-block	1 (2.1%)
RRT/plasmapheresis	9 (19.1%)
Not available	6
Outcomes	
Complete or partial remission	41 (91.1%)
No progression	4 (8.9%)
Not available	8

ANCA: antinuclear cytoplasmic antibody, GN: glomerulonephritis; MCD: minimal change disease; FSGS: focal segmental glomerulosclerosis; RRT: renal replacement therapy.

### 2.1. Pauci-immune GN and renal vasculitis

Pauci-immune GN and renal vasculitis are normally regarded as the common types of glomerular lesions related to ICI use. Compared with other types of glomerular diseases, pauci-immune GN and renal vasculitis have a relatively higher incidence in female (*n* = 6) while the total number of female is 11 (Table 1). The symptoms were relatively severe, the Scr level peaked at 4.68 ± 1.7 mg/dL, and renal pathology in severe cases revealed focal segmental necrotizing glomerulonephritis and focal global sclerosis [18,30]. Moreover, the treatment was complex (Supplementary Table 1). High-dose methylprednisolone was used in 6 cases, including 2 receiving cyclophosphamide and 1 receiving mycophenolate together [18,27,30,40]. Rituximab was used in 5 patients [30], and 6 cases were treated with renal replacement therapy (RRT) or hemodialysis [18,20,30,40]. The prognosis was poor. Most patients achieved only a partial remission, and renal function could not be fully restored. Only four cases reported a positive anti-neutrophil cytoplasmic antibodies (ANCA) serology (4/15), including 2 received pembrolizumab [18,22], 1 rceived tremelimumab [30], and 1 received ipilimumab and pembrolizumab [17]. ANCA positivity did not apparently show an association to a certain class of ICI used or a poorer renal outcome (Supplementary Table 1).

### 2.2. Podocytopathies

Podocytopathies are the second most reported glomerular diseases correlated with ICIs, which included 12 cases of MCD and 2 cases of focal segmental glomerulosclerosis (FSGS). Our statistics suggested that most cases (8/12) were related to anti-PD-1 antibodies (including pembrolizumab and nivolumab). Only one case was treated with combination medication. Glutsch et al. [28] reported a case with MCD caused by pembrolizumab monotherapy and revealed that the patient deeply responded to well-tolerated ipilimumab and nivolumab when given simultaneously. Most patients discontinued ICI, and all underwent systemic steroid treatment (prednisone 0.5–2 mg/kg/d) with completely or partially remitted proteinuria. All the patients were not treated with RRT or hemodialysis (Supplementary Table 2). Generally, compared with Pauci-immune GN and renal vasculitis, the prognosis of nephrotic syndrome (NS) related to ICIs is well.

### 2.3. Immune-complex GN

The incidence of immune-complex GN associated with ICIs therapy was also high, with 10 related cases consisting of 6 cases of IgAN [11,20,35,36,38], one Lupus Nephritis [13], one immune mediated glomerulonephropathy with IgA and C3 deposits [15], one immune mediated glomerulonephropathy with only IgM deposits [25], and one immune mediated glomerulonephropathy with deposits of C3 (2+-3+) and IgG (1+) [23]. For different types of immune-complex GN, various clinical manifestations were observed. The Scr increased from 0.79 to 10.08 mg/dL. Compared with the other four immune-complex GN, the incidence of IgAN was relatively high (6/10), and the clinical manifestation was relatively mild. Five patients with IgAN discontinued ICIs, and only three were treated with steroids. Only one patient presented with severe symptoms and was treated with mycophenolate mofetil (MMF) and infliximab [11]. After the treatment, proteinuria received complete or partial remission. For the other 4 cases of immune-complex GN, 2 received high-dose methylprednisolone, and 1 received hemodialysis together. All the patients received complete or partial remission.

## 3. Mechanisms of glomerular diseases related to ICIs use

CTLA-4, a key negative regulator of T cell activation, is located on the T cell surface and prevents T cell activation by outcompeting CD28 for its ligand, B7, thereby inhibiting T cell co-stimulation. PD-1, which protects against autoimmune diseases, is a cell surface receptor on the T cell. PD-L1, a protein expressed on cancer cells, can bind to PD-1 and inhibit the T cell from attacking the cancer cell [2]. The expressions of CTLA-4 and PD-1 in the kidney have no specificity. However, it was discovered that PD-L1 was present in distinct renal compartments in nephrotoxicity related to ICI therapy [46]. Besides, Hakroush et al. revealed that PD-L1 is frequently expressed in various renal pathologies independent of ICI therapy and could potentially be a pre-requisite for susceptibility to develop AKI and deleterious immune-related acute interstitial nephritis (AIN) [47].

The mechanisms and manifestations of irAEs caused by ICIs are based on the type of ICI therapy. CTLA-4 inhibitors can induce many cellular changes, such as T-cell activation and proliferation, survival of impaired CD4+ CD25+ regulatory T cell (Treg cell), and an increase in the number of type 17 T helper cells. Besides, cross-reactivity between anti-tumor T cells and antigens is induced in healthy cells with the production of autoantibodies. PD-1 and PD-L1 inhibitors lower the survival and inhibitory function of Treg cells while increasing cytokine production [7,11]. It was revealed that in PD‐1‐deficient mice, autoimmune diseases were commonly concentrated in the organs, while in CTLA-4 deficient mice, autoimmune diseases were wide, systemic, and fetal [48]. Extensive use of ICIs with various doses lead to renal toxicity. The use of ICI time was from 18 days to 2 years, and the shortest dose is 1 cycle [27,28]. Considering that the T-cell autoreactive clones causing these irAEs may last for long after the treatment ceases, irAEs may even remain for several years following the treatment. Renal vasculitis (28.3%) and podocytopathies (26.4%) were the two types that were most frequently reported (Table 1), although it remains unclear why these two have a high incidence rate. T cells, which include regulatory T cells, participate in the emergence and adjustment of the ANCAs and can induce tissue injury. Researchers have proved that abnormal PD-1 and CTLA-4 levels remarkably affect the pathophysiology of vasculitis [49,50]. ICI use is related to various types of renal vasculitis, most of which are ANCA negative and present severe AKI. PD-1 may be capable of lowering autoimmunity and promoting self-tolerance to ensure that endogenous or exogenous stimuli cannot be overactivated when physiological conditions are normal [51]. Besides, PD-1 can regulate T cell activation to maintain immune activity, thus preventing T cells from being affected by kidney events due to adverse immune responses [52]. In a study on mice withdriamycinn nephropathy, PD-1 blockade aggravated kidney injury in terms of histopathology and function, reflecting that PD-1 may play a protective role in chronic renal disease [53]. The mechanism of ICIs-induced glomerular diseases is still unclear. The main hypotheses are as follows:

### 3.1. Nonspecific activation of the immune system

ICIs enhance T cell immune function without selectivity between tumor tissue and normal tissue, down-regulate its tolerance to its antigens, and hence cause nonspecific activation of the immune system. Glomerular disease is induced by the ‘reprogramming’ of the immune system [54]. CTLA-4 deficiency has been discovered in both humans and mice to induce autoimmune lymphocytic syndrome through the activation of self-antigen-specific T cells [55]. Additionally, PD-1 knockout led to the generation of immune-complex GN [56], revealing the crucial significance of the PD-1 signaling pathway to the minimization of renal inflammation under the mediation of T cells.

### 3.2. Immune-complex-mediated kidney injury

ICIs induce the production of autoimmune antibodies, leading to the deposition of antibody-antigen complexes in the kidney. Lute et al. generated human CTLA4 gene knock-in mice. They used them to compare a panel of anti-human CTLA-4 antibodies for their ability to induce autoimmunity. The group suggested that the treatment with anti-CTLA4 antibodies revealed circulating anti-double-stranded DNA antibodies as well as glomerular IgG and C3 deposits [57]. Fadel et al. reported the first case of glomerulonephritis occurred by ipilimumab, and the kidney biopsy revealed lupus nephritis. Ipilimumab withdrawal was followed by the appearance and the regression of circulating anti-double-stranded DNA antibodies [13].

### 3.3. Cytokines release

ICIs enhance proinflammatory cytokines/chemokines release in kidney tissue, further enhancing podocyte foot-process effacement. It has been hypothesized that by adopting therapies modulating T lymphocyte regulation, cytokine release was increased, thereby influencing VPF/VEGF secretion and promoting subsequent proteinuria [10]. Additionally, ICIs can expand T cells while promoting the interaction between T cells and B cells, leading to the subsequent production of cytokines interferon-gamma and IL-21, both of which are related to ANCA vasculitis [58]. Besides, serum levels of cytokines such as CXCL-10, TNF alpha, and IL-6 were elevated in patients developing TIN after receiving the treatment of ipilimumab and nivolumab [59].

### 3.4. Cross-reactivity with off-target kidney tissues

ICIs can assist in forming new or reactivated T cells against tumor antigens (Ags) which are capable of cross-reacting with off-target kidney tissues [60]. Johnson et al. reported the development of myocarditis in a patient who received treatment with ICIs. Myocardial cells, skeletal muscle cells, myocardium/skeletal muscle cells, and tumor cells showed diffuse infiltration of CD4+ T cells and CD8 + T cells [61].

### 3.5. Complement alternative pathway

The complement alternative pathway could be a target of autoimmune dysregulation (AP) caused by ICIs. Ville et al. presented a case of C3 GN related to pembrolizumab use. The patient was screened for a wide range of autoantibodies with all negative except for anti-C3b [26].

**Figure 1.**
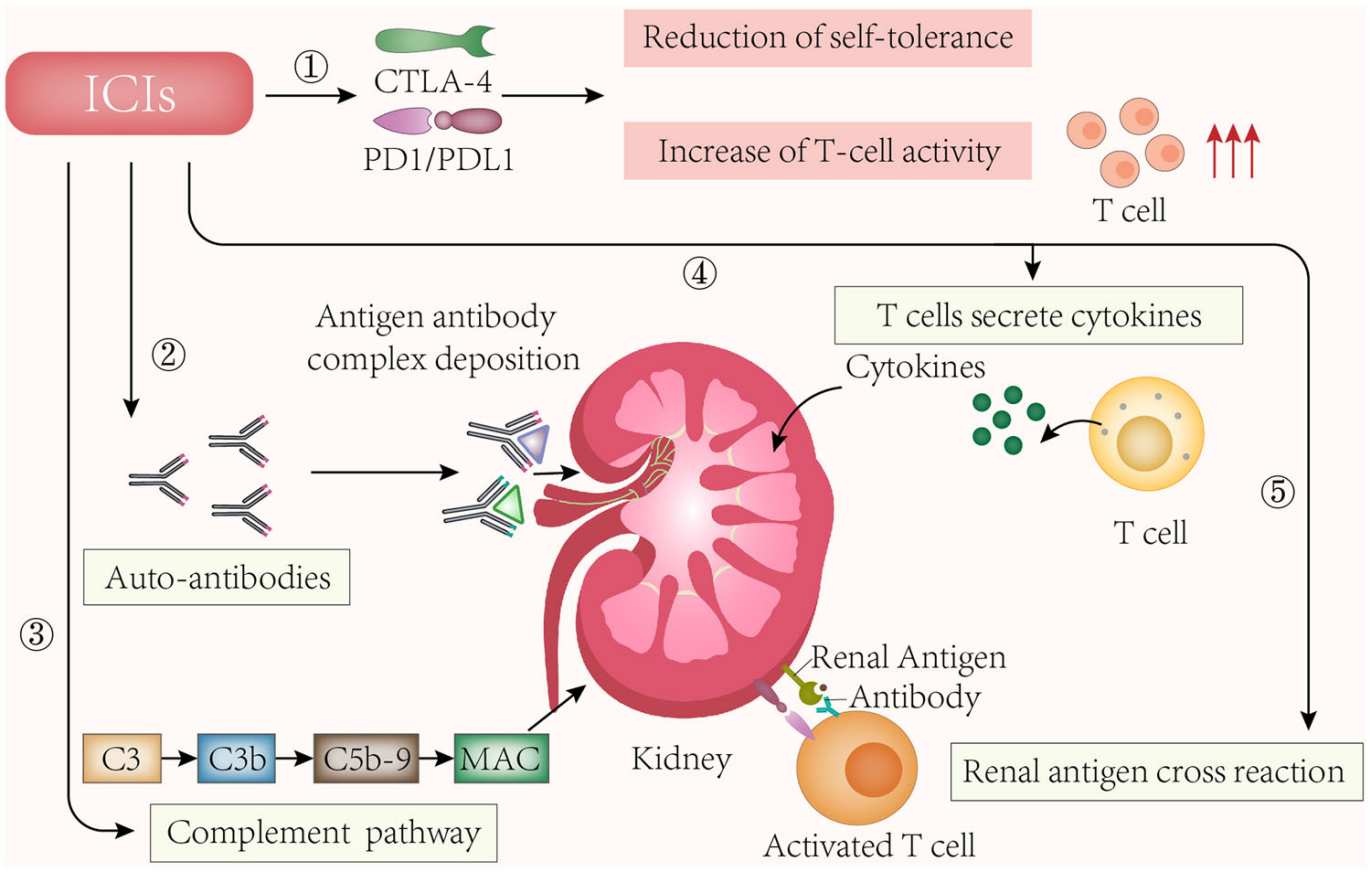
Potential mechanisms of ICIs induced glomerular diseases. ICIs: immune checkpoint inhibitors; CTLA-4: cytotoxic T lymphocyte-associated antigen 4; PD1: programmed death 1 protein; PDL1: programmed death-ligand 1; MAC: membrane attack complex. Nonspecific activation of the immune system, induce the production of autoimmune antibodies and antibody-antigen complexes deposit in the kidney, increase proinflammatory cytokines/chemokines in kidney tissue; cross-reactivity between anti-tumor T cells and antigens is induced on healthy cells, and form membrane attack complex by complement alternative pathway are potential mechanisms of ICIs induced glomerular diseases.

The diagram of the plausible mechanism is illustrated in Figure 1.

## 4. Treatment and management of ICIs-related glomerular diseases

The treatment of ICIs-related kidney injury is aggressive. Notably, Scr level was normal in a few patients, such as MCD, and early manifestations may present with edema, oliguria, or elevation of blood pressure [10,14]. These patients should be closely observed, and a kidney biopsy should be performed if necessary. Treatments of ICIs-associated glomerular diseases are challenging than TIN, and there is no unified treatment protocol. Renal pathological changes and the possibility of malignant tumor progression should be considered when creating a treatment plan. The nephrologist and the oncologist must develop a treatment plan jointly. Routine treatments include ICIs withdrawal and steroid therapy. The treatment plan is mainly based on renal pathological changes, while AKI severity can also be used as a reference index [62].

### 4.1. Icis discontinued

In most of the 53 cases, ICIs were discontinued immediately. Irrespective of whether the treatment continues, the positive effect remains. The benefit of stopping exposure to ICIs is significant. Kishi et al. [20] discovered a case where a patient developed IgAN after being treated with nivolumab. Drug withdrawal without steroids treatment was followed. When the report was published, recurrence of lung cancer was not observed, and proteinuria improved.

However, it is controversial whether ICI therapy should be discontinued when glomerular disease occurs. For patients with mild renal pathologic changes and Scr of grade 1–2, ICI therapy may be considered for persistent use, requiring close monitoring of renal function, electrolytes, and urinary abnormalities. Additionally, the combination therapy with corticosteroid or rituximab might allow continued use of the ICIs despite the occurrence of renal irAEs [25,34]. Irifuku et al. presented a case of a patient with RCC who developed immune-complex GN with a Scr level of 3.09 mg/dL approximately 4 months after initiation of nivolumab. The group initiated high-dose prednisolone therapy with nivolumab, thereby contributing to improved renal function and achieving complete remission of proteinuria [25]. Lin et al. provided a case of reactivated autoimmune MN following ICI therapy responsive to rituximab. The patient received five cycles of nivolumab continuously following rituximab therapy with improved kidney function [34]. Thus, continuing ICI therapy could be considered with close monitoring when ICI therapy plays a key role in the survival benefit. Combination therapy with steroids or rituximab might be beneficial for continued ICI therapy.

### 4.2. Steroid therapy

Steroid therapy was employed in most ICIs-related glomerular diseases. The effect of steroid therapy is effective and remarkable [9]. All of the cases of MCD and FSGS were treated with glucocorticoids and achieved complete or partial remission. Regular doses of prednisone or methylprednisolone were commonly used, with a dose of 0.5–2 mg/kg/d according to the AKI severity and renal pathology, tapered off over 6 to 26 weeks. It is allowed to use a pulsed dose of steroids for patients with resistant renal dysfunction, severe clinical manifestations, and severe pathological changes in renal biopsy, involving severe renal vasculitis, cell crescent formation, and glomerular necrosis. The dose of methylprednisolone is 1 g intravenously daily for 3 days. Notably, a rapid reduction in the dose of steroids may lead to a relapse of the disease. For steroid-dependent patients, steroids combined with immunosuppressive treatments may have an improved effect [9]. Besides, the use of glucocorticoids did not affect the response and prognosis of anti-tumor therapy for patients with melanoma [7]. Theoretically, corticosteroids may reduce T-cell activity and could affect response to ICI, however, it suggested that short course of corticosteroid played no effect on the prognosis. Sorial et al. declared that corticosteroids should be used with ICIs when indicated and should not be withheld due to concerns about worse outcomes [63].

### 4.3. Mycophenolic therapy (MMF)

MMF is adopted to prevent organ transplant rejection and treat autoimmune diseases like systemic lupus erythematosus and steroid-resistant NS. With respect to the renal irAEs, MMF would be adopted for patients with hormone insensitivity or drug resistance. Findings about MMF remain controversial. It is deleterious for TIN patients as it can cause pancytopenia and fatal septic complications [64], while it is beneficial for FSGS patients [12]. A total of 3 patients were treated with MMF; 1 died of respiratory failure before remission, while the other 2 achieved partial remission [11,12,40]. It was suggested that when corticosteroids have an insufficient effect, MMF may be a helpful addition in treating nivolumab-induced NS [12].

### 4.4. Rituximab therapy

Considering the specificity in targeting B-lymphocytes, rituximab may provide a good treatment option for glomerular disease and cannot directly impact cancer therapy. Rituximab should be considered for treating ICI-induced vasculitis. Mamlouk et al. presented five cases of ICI-induced vasculitis, and all the cases received the treatment of prednisone and rituximab [11]. Moreover, the patients exhibited good tolerance, and their kidneys recovered partially or completely without relapse. The malignancy risk was discovered to be lower in rituximab-treated ANCA-associated vasculitis patients relative to patients treated with cyclophosphamide; rituximab treatment did not lead to an increase in malignancy risk relative to the general population [65]. Contrary to cyclophosphamide which is used to reduce depleting T cells, rituximab is capable of disrupting the pathogenic B cell/CD8+ T cell axis. Thus, there is less production of T cell cytokine, the renal endothelial damage reduces subsequently, and there is no hindrance in ICI anti-tumor activity [66].

### 4.5. Renal replacement therapy (RRT)

Supportive care should be initiated when severe and persistent AKI occurs. It was reported that the RRT rate is 9% in all ICI-related AKI patients [39], while 25% of patients with glomerular diseases needed RRT [67]. Plasma exchange has been used in severe renal vasculitis [30]. However, despite using plasma exchange, the incidence of death or ESKD among patients with severe ANCA-associated vasculitis was not reduced [68].

### 4.6. Other therapy

Angiotensin-converting enzyme inhibitor (ACEI) treatment and diuretics may be initiated with volume control in NS [42]. Cyclophosphamide may be applied in renal vasculitis [18], while its toxic and side effects causing absolute lymphopenia in T cells and B cells should be considered. Despite the wide application of anti-TNF alpha drugs in digestive irAEs, no indications could be found for renal complications. In these reported cases, only one patient received TNFα-blockade, but the renal function did not recover [40]. Besides, targeting CD28-CD80 or CTLA-4 can help reverse certain autoimmune phenomena by using the immune checkpoint blockade. Nonetheless, there was no similar observation in kidney diseases.

## 5. Relapsing glomerular diseases after ICIs use

Over one-third of patients suffering from preexisting rheumatic or autoimmune diseases (such as lupus, rheumatoid arthritis, and inflammatory bowel disease) have experienced flares of their prior disorder ascribed to the use of ICIs for malignancy [69]. Anti-PD-1/PD-L1 agents more easily led to disease flares relative to CTLA-4 blockade (62% vs. 36%) [67]. For these reported ICIs-related glomerular diseases, only two patients had a history of nephropathy before ICIs use, both of which were MN. A total of 4 cases of MN were reported, two of which were of major interest. Lin et al. [34] reported the first case of ICIs-associated MN reactivation in a patient who received anti-PD1 therapy. The patient had about 14 years of primary MN history. His MN relapsed only once in 2017. Kim [42] described a case of NS relapse in a patient with a history of MN when receiving PD-1 inhibitor therapy for treating lung cancer. MN recurrence was considered for the reappearance of proteinuria after the use of nivolumab/durvalumab. As speculated, by relying on prolonged ICIs therapy, immune tolerance could escape, and the susceptibility could increase to trigger the MN relapse in the patient. Hence, using ICIs may lead to the recurrence of previous kidney disease. However, the number of related cases is limited, and the probability and severity of the recurrence of glomerular diseases need further investigation.

## 6. Rechallenging ICIs therapy with ICIs-associated glomerular diseases

Among the reported cases of ICIs-associated glomerular diseases, a total of five cases (10.2%) were found with a rechallenge of ICIs therapy [10,23,28,31,38]. Rechallenge may involve either the use of the same drug or a different ICI. Saito et al. presented a case of a patient who exhibited a remarkable response to pembrolizumab and underwent re-administration of pembrolizumab and prednisolone after recovery from ICI-related MCD; there were no adverse effects such as NS recurrence [31]. In the report of Glutsch et al. [28], a patient with MCD due to pembrolizumab monotherapy was re-exposed to immunotherapy combining ipilimumab and nivolumab because of a disease progression. Fortunately, nephrotoxicity was tolerated with deep partial remission. The rechallenging may receive a good result with tolerable nephrotoxicity. However, ICI-related renal iRAEs may reappear, and ICI therapy might be withdrawn permanently. Kitchlu et al. [10] reported a case of a patient who developed ipilimumab-related MCD and restarted ipilimumab as salvage therapy. However, NS recurred, and ipilimumab therapy was again discontinued. The rechallenge with ICI therapy is controversial and requires balancing kidney disease, AKI severity, and cancer progression. Particularly, kidney injury may lead to higher mortality [39]. It is difficult to offer treatment guidance because of very few reports of rechallenging ICI therapy with glomerular diseases. Our study suggested that the rechallenge of ICIs should be considered for cancer progression or as salvage therapy. Rechallenging ICI therapy with steroids may be beneficial. Meanwhile, renal function and the patient’s condition must be closely monitored.

## 7. Conclusions

In conclusion, researchers have reported various pathologies of glomerular disease associated with ICIs. The most frequently reported were pauci-immune GN, podocytopathies, and immune-complex GN. Anti-PD1 antibodies were the most widely used ICIs. Various ICI-causing renal manifestations suggested that it is necessary to further elucidate its complicated mechanisms. The time of AKI or proteinuria occurrence is inconsistent and even occurs months after discontinuing ICIs. Therefore, regular monitoring of urine routine and Scr is required. It is emphasized in this paper that kidney biopsy is suitable for diagnosing ICIs-induced kidney disease. Discontinuation of ICIs and glucocorticoid treatment can improve the renal outcome of most patients. The treatment should be personalized according to the degree of renal pathology, Scr, and tumor progression to obtain a good prognosis. Rechallenge with ICIs should be considered for cancer progression or as salvage therapy. This requires the collaboration of several specializations, such as the oncology, nephrology, and urological surgical fields. Proper biomarkers for toxicity prediction should be discovered to improve the management of patients with ICI therapy [70]. However, factors for predicting kidney toxicity after treatment with ICIs are deficient. The targeted therapy and predictive factors for ICIs-related renal diseases should be further investigated in the future.

## Supplementary Material

Supplemental Material
