# Supplementary material for: Glomerular diseases after immune checkpoint inhibitors use: What do We know so far?
Source: Ren Fail. 2022 Nov 24;44(1):2046–55. doi: 10.1080/0886022X.2022.2147439 (PMC9704066; doi:10.1080/0886022X.2022.2147439)
Supplement: Supplemental Material [file IRNF_A_2147439_SM5647.pdf]

Supplementary Table 1. Summary of published reports of renal vasculitis and pauci-immune glomerulonephritis associated with ICIs

Year	Author	Sex	Age	Tumor type	ICIs tharepy	Glomerular diseases	Peak SCr (mg/dL)	Cycles or times of ICIs use	ICIs discontinued	steroids	HDMP	immunosuppressant	CRRT/hemodialysis	outcomes
2016	van den Brom [17]	F	56	metastatic melanoma	ipilimumab + pembrolizumab	granulomatosis with polyangiitis PR3-ANCA+	NA	ipilimumab 4cycles+ pembrolizumab 1cycle	Y	Y	Y	cyc 150mg/d	N	partial
2017	Heo [18]	M	56	thymic epithelial tumor	pembrolizumab	ANCA–associated rapid progressive glomerulonephritis ANCA 1:160	4.49	5	Y	Y 2 mg/kg/d	Y 0.5g/d 3d	cyc 150mg/d	hemodialysis	partial
2018	Cho [22]	NA	NA	Thymoma	pembrolizumab	renal vasculitis, ANCA+	NA	NA	Y	Y	N	N	hemodialysis	NA
2019	Lemoine [29]	M	70	anal metastatic melanoma	nivolumab 1 year + ipilimumab 2months	juxtaglomerular granulomatous arteritis，ANCA-	5.8	3	Y	Y 1 mg/kg/d	N	N	N	partial
2019	Gallan [27]	M	68	metastatic anal mucosal melanoma	nivolumab	renal vasculitis wtih focal global glomerular sclerosis, ANA1:80 ANCA undetected	1.7	2	NA	NA	N	N	N	NA
2019	Gallan [27]	F	71	metastatic lung adenocarcinoma	pembrolizumab	pauci-immune glomerulonephritis, ANCA-	NR	10 months	Y	Y	Y	N	N	complete
2019	Gallan [27]	F	75	metastatic non–small-cell lung carcinoma	nivolumab	renal vasculitis, Granulomatous, ANCA-	6.1	2 years	Y	Y	N	N	N	partial

2019	Gallan [27]	F	63	metastatic choroidal melanoma	nivolumab	renal vasculitis, ANCA-	5.1	14 weeks	Y	Y	Y	N	N	partial
2020	Mamlouk [30]	M	41	Metastatic non-small cell lung cancer	nivolumab	renal vasculitis，ANCA-	4.52	5	Y	Y 1 mg/kg/d	N	Rituximab (1 dose)	N	complete
2020	Mamlouk [30]	M	70	metastatic renal cell carcinoma	tremelimumab	renal vasculitis（acute focal segmental pauciimmune necrotizing glomerulonephritis）MPO1:160	4.58	2	Y	Y 2 mg/kg/d	N	Rituximab (4dose)	plasmapheresis	partial
2020	Mamlouk [30]	F	69	Metastatic uveal melanoma	Ipilimumab + nivolumab	renal vasculitis，ANCA-	4.96	3	Y	Y 1mg/kg/d	N	Rituximab (1 dose)	N	complete
2020	Mamlouk [30]	M	60	Liposarcoma	nivolumab	renal vasculitis，ANCA-	7.53	2	Y	Y	Y	Rituximab (2 dose)	RRT+ plasmapheresis	partial
2020	Mamlouk [30]	F	50	Metastatic uveal melanoma	ipilimumab + nivolumab	renal vasculitis, ANCA-	5	2	Y	Y	Y	Rituximab (a biweekly dose)	RRT + hemodialysis	partial
2020	Person [40]	M	55	melanoma of the anal mucosa multifocal hepatic metastases	nivolumab + ipilimumab (followed by pembrolizumab	Vasculitis, Granuloma	1.7	1	Y	Y	Y 0.2g/d	Mycophenolate/TNFα-block	RRT	NO
2020	Cortazar [39]	NA	NA	NA	NA	pauci-immune crescentic GN, ANCA-	NA	NA	NA	NA	NA	NA	NA	NA

ANCA, antineutrophil cytoplasmic antibody; Cyc, Cyclophosphamide; F, female; HDMP, High dose methylprednisolone; ICI, immune checkpoint inhibitor; M, male;

NA, not available; NR: normal range; RRT, renal replacement therapy.

Supplementary Table 2. Summary of published reports of MCD and FSGS with ICIs

Year	Author	Sex	Age	Tumor type	ICIs tharepy	Glomerular diseases	Peak SCr (mg/dL)	Cycles of ICIs use	ICIs discontinued	steroids	HDMP	immunosuppressant	Outcome
2016	Bickel [14]	M	62	malignant pleural mesothelioma	pembrolizumab	MCD	NR	2	Y	Y 1mg/kg	N	N	complete
2016	Kidd [16]	M	55	metastatic melanoma	ipilimumab	MCD	5.2	NA	Y	Y 2mg/kg	N	N	complete
2017	Kitchlu [10]	M	43	Hodgkin lymphoma	pembrolizumab	MCD	3.93	2	Y	Y 2mg/kg	N	N	partial
2017	Kitchlu [10]	M	45	metastatic melanoma	ipilimumab	MCD	NR	4	Y	Y 1mg/kg	N	N	complete
2017	Daanen [12]	M	62	papillary renal cell	nivolumab	FSGS	2.95	4	Y	Y	Y 1g/d 3d	Mycophenolate	partial
2018	Gao [19]	M	40	Hodgkin lymphoma	SHR-1210	MCD	NR	3	Y	Y 1mg/kg	N	N	complete

2019	Izzedine	NA	NA	Melanoma	pembrolizumab	MCD	NA	NA	NA	NA	NA	NA	NA
2019	Izzedine [24]	NA	NA	Ileal neuroendocrine tumor	pembrolizumab	MCD	NA	NA	NA	NA	NA	NA	NA
2019	Mamlouk [11]	M	74	renal cell carcinoma	nivolumab	FSGS	2.73	14 months	Y	Y 0.8mg /kg/d	N	N	partial
2019	Saito [31]	M	79	lung adenocarcinoma	pembrolizumab	MCD	NR	7	Y	Y 40mg/d	N	N	complete
2019	Glutsch [28]	M	68	melanoma	pmbrolizumab , rechange with ilimumab plus nivolumab	MCD	2.86	1	Y	Y 100mg/d	N	N	partial
2020	Vaughan [37]	M	57	squamous cell carcinoma of the tongue	nivolumab	MCD	2.26	2	Y	Y	N	N	NO
2021	Toda [43]	M	75	non small cell lung cancer	durvalumab （PD -L1 inhibitor）	MCD	NA	4	Y	Y	N	N	complete
2020	Cortazar [39]	NA	NA	NA	NA	MCD	NA	NA	NA	NA	NA	NA	NA

FSGS: focal segmental glomerulosclerosis; HDMP, High dose methylprednisolone; ICI, immune checkpoint inhibitor; M, male; MCD, minimal change disease; NA, not available; NR: normal range; RRT, renal replacement therapy

Supplementary Table 3. Summary of published reports of combination therapy of ICIs

Year	Author	Sex	Age	Tumor	ICIs	Glomerular diseases	Peak SCr (mg/dL)	ICIs discontinued	steroids	Other therapy	Outcome
2016	van den Brom [17]	F	56	melanoma	ipilimumab 4cycles+pembrolizumab 1cycle	granulomatosis with polyangiitis (PR3-ANCA+)	NA	Y	Y	HDMP + Cyc 150mg/d	partial
2019	Lemoine [29]	M	70	melanoma	nivolumab 1 year +ipilimumab 2months	juxtaglomerular granulomatous arteritis	5.8	Y	Y1mg/kg/d	N	partial
2019	Glutsch [28]	M	68	melanoma	pembrolizumabip 1cycle，rechange with ilimumab + nivolumab	MCD	2.8	Y	Y100 mg/d	N	partial
2019	Mamlouk [11]	M	69	melanoma	ipilimumab and nivolumab	IgA nephropathy	2.4	Y	Y0.5 mg/kg/d	N	complete
2019	Ashour [23]	M	68	melanoma	pembrolizumab 3cycles, rechange with nivolumab with one-year interval	immune complex–mediated GN	5.5	Y	Y 2 mg/kg/d	N	partial

2020	Mamlouk [30]	F	69	melanoma	ipilimumab and nivolumab	renal vasculitis，ANCA-	4.9	Y	Y1mg/kg/d	Rituximab (1 dose)	complete
2020	Mamlouk [30]	F	50	melanoma	ipilimumab and nivolumab	renal vasculitis,ANCA-	5	Y	Y	HDMP + Rituximab (a biweekly dose) + RRT with hemodialysis	partial
2021	Person [40]	M	55	melanoma	nivolumab and Ipilimumab 1cycle，followed by pembrolizumab	TMA, Vasculitis, Granuloma	1.7	Y	Y	HDMP + Mycophenolate + TNFα-block + RRT	NO

ANCA, antineutrophil cytoplasmic antibody; Cyc, Cyclophosphamide; F, female; HDMP, High dose methylprednisolone; ICI, immune checkpoint inhibitor; M, male; NA, not available; RRT, renal replacement therapy
